# Proteomic Analysis of Seedling Roots of Two Maize Inbred Lines That Differ Significantly in the Salt Stress Response

**DOI:** 10.1371/journal.pone.0116697

**Published:** 2015-02-06

**Authors:** Dezhou Cui, Dandan Wu, Jie Liu, Detao Li, Chunyan Xu, Song Li, Peng Li, Hua Zhang, Xu Liu, Chuan Jiang, Liwen Wang, Tingting Chen, Huabang Chen, Li Zhao

**Affiliations:** 1 The State Key Laboratory of Plant Cell and Chromosome Engineering, Institute of Genetics and Developmental Biology, Chinese Academy of Sciences, Beijing, P.R. China; 2 Institute of Crops Sciences, Shandong Academy of Agricultural Sciences, Jinan, Shandong, P.R. China; 3 School of Life Science, Shandong University, Jinan, Shandong, P.R. China; 4 The State Key Lab of Crop Biology, College of Agriculture, Shandong Agricultural University, Tai’an, Shandong, P.R. China; The University of Melbourne, Australia

## Abstract

Salinity is a major abiotic stress that limits plant productivity and quality throughout the world. Roots are the sites of salt uptake. To better understand salt stress responses in maize, we performed a comparative proteomic analysis of seedling roots from the salt-tolerant genotype F63 and the salt-sensitive genotype F35 under 160 mM NaCl treatment for 2 days. Under salinity conditions, the shoot fresh weight and relative water content were significantly higher in F63 than in F35, while the osmotic potential was significantly lower and the reduction of the K^+^/Na^+^ ratio was significantly less pronounced in F63 than in F35. Using an iTRAQ approach, twenty-eight proteins showed more than 2.0- fold changes in abundance and were regarded as salt-responsive proteins. Among them, twenty-two were specifically regulated in F63 but remained constant in F35. These proteins were mainly involved in signal processing, water conservation, protein synthesis and biotic cross-tolerance, and could be the major contributors to the tolerant genotype of F63. Functional analysis of a salt-responsive protein was performed in yeast as a case study to confirm the salt-related functions of detected proteins. Taken together, the results of this study may be helpful for further elucidating salt tolerance mechanisms in maize.

## Introduction

Salinity is a major abiotic stress that affects plant growth and yield throughout the world [1–3]. More than 830 million hectares of land, which account for over 6% of the world’s total land area, have been affected by salinity [4]. Maize (*Zea mays* L.) plays an important role in global food security and economic development. Unfortunately, maize is not a salt-tolerant crop. Therefore, improving salt tolerance has become important for maize production.

Under salt stress conditions, the establishment of healthy seedlings is extremely important for maize plant subsequent development. Roots are the primary sites of salinity perception, and salt sensitivity in roots limits the productivity of the entire plant [5]. Therefore, obtaining a better understanding of salt-responsive mechanisms in seedling roots is critical for improving plant salt tolerance.

To better understand the molecular mechanisms of plant salt tolerance, large-scale transcriptomic analyses have been employed in the roots of numerous plant species. However, transcriptome profiling has limitations because mRNA levels are not always correlated to those of corresponding proteins due to post-transcriptional and post-translational modifications [6–8]. Elucidating changes at the protein level is essential for studying salt stress responses in plants. Proteomic analysis provides new insights into plant responses to salt stress at the protein level[9,10]. Recent advances in proteomics have made it possible to perform large-scale studies to help elucidate salt tolerance mechanisms in roots. To date, more than 905 salt-responsive proteins have been identified in roots from 14 plant species, such as *Arabidopsis*, rice, wheat, soybean, tomato, and barley [10]. However, relatively few such studies have been performed in maize. Zörb et al. studied proteomic changes in maize roots after a short-term adjustment to saline growth conditions [11]. A set of phosphoproteins were detected. Nevertheless, the application of 25 mM NaCl in this study appears to be relatively low for salt stress treatment, which may have led to the identification of a small number of salt-responsive proteins.

In the current study, to study maize salt responses at the protein level, we examined two contrasting maize inbred lines that showed significantly different phenotypes and physiology under salt stress. We conducted a comparative proteomic analysis of these two lines under 160mM NaCl treatment for 2 days. In addition, we examined the functions of one salt-responsive protein in yeast. The results of this study may be helpful for further salt tolerance studies in maize.

## Materials and Methods

### Plant materials and NaCl treatment

A total of 162 maize (*Zea Mays* L.) inbred lines (S1 Table) were used for salt tolerance screening in both the field and in hydroponic solutions. For field screening, 15 seeds were sown for each inbred line, and three replicates were conducted. Seeds were planted in soil compartment in which the NaCl concentration was adjusted to 0.3% (w/v) in Nanpi County, Hebei province, China. For hydroponic selection, five seedlings were used per line, and three replicates were performed. Seedlings were grown hydroponically in Hoagland’s full-strength nutrient solution until the third leaf was fully developed. The solution was aerated continuously with an electric pump and replaced every 2 days. Then, half of the seedlings were cultured in nutrient solution containing a final concentration of 160 mM NaCl while the remaining (control) samples were grown in solution lacking NaCl. The most salt-tolerant genotype and the most salt-sensitive genotype were selected for further analysis. For proteomic analysis, roots from ten plants were harvested and washed with distilled water for three times before being immersed into liquid nitrogen after 2 days of NaCl treatment from control (untreated) and treated samples; the samples were stored at -80°C for further use. Two independent biological replicates were conducted for proteomic analysis to validate the results.

### Measurement of physiological parameters

Physiological responses to salinity stress were evaluated by measuring fresh weight, relative water content (RWC), osmotic potential, and relative electrolyte leakage (REL) in shoots, as well as cation content in both shoots and roots after salt treatment. To examine physiological changes in the seedlings, the average values from ten seedlings were calculated for each genotype, and five independent biological replicates were conducted. Plant shoot fresh weight and RWC were measured once per day for 6 consecutive days after salt treatment. Osmotic potential, REL, and cation content were measured after 2 days of treatment. For RWC, leaf fresh weight was measured immediately after the leaves were cut off the seedlings. Then, the sample was immersed in deionized water and incubated at 4°C overnight. The weight of the sample represented its rehydrated weight. Finally the sample was completely dried in an oven and its dry weight was calculated. RWC was calculated as (fresh weight—dry weight) / (rehydrated weight—dry weight). Leaf osmotic potential was measured with a vapor pressure osmometer (Vapro 5600, Wescor, Logan, UT, USA). Fresh leaves were obtained and soaked into deionized water for 6 h. The surface water was removed, and the leaves were frozen at -20°C for 2 h. The leaves were then thawed and pressed to obtain cell sap, which was subsequently analyzed for osmolarity (Os; mmol kg^-1^); osmotic potential (Mpa) = -Os × 2.58 × 10^–3^ [12,13]. The REL assay was conducted according to Liu et al. [13]. To measure cation contents, the roots were washed with distilled water for three times, plant shoots and roots were dried at 80°C and digested with 1% acid mixture (nitric acid: perchloric acid = 4:1). Na^+^ and K^+^ contents were analyzed using an Eppendorf flame photometer (Eppendorf, Hamburg, Germany).

### Protein extraction

Total proteins were extracted from roots according to Lan et al. [14]. Total protein concentrations were determined using a Bradford Protein Assay Kit (GE Healthcare, Pittsburgh, PA, USA) according to the manufacturer’s instructions and the protein samples were stored at -80°C.

### Protein digestion and iTRAQ labeling

Total proteins (100 μg samples) were digested with Trypsin Gold (Promega, Madison, WI, USA) at a ratio of protein: trypsin = 30:1 at 37°C for 16 h. After digestion, peptides were dried by vacuum centrifugation and reconstituted in 0.5 M TEAB. Labeling was performed according to the manufacturer’s protocol for iTRAQ (AB Sciex, Foster City, CA USA) with minor modifications. In brief, one unit of iTRAQ reagent (defined as the amount of reagent required to label 100 μg of protein) was thawed and reconstituted in 70 μL isopropanol. The control replicates were labeled with iTRAQ tags 113 and 114 for the salt-sensitive genotype and, 115 and 116 for the salt-tolerant genotype. The 160 mM NaCl treated replicates were labeled with tags 117 and 118, 119 and 121 for the salt-sensitive and-tolerant genotypes, respectively. The labeling reactions were incubated at room temperature for 2 h. Two technical replicates were performed.

### LC-ESI-MS/MS analysis

LC-MS/MS analysis was performed on an LC-20AD nanoHPLC (Shimadzu) connected to an LTQ-Orbitrap Velos hybrid mass spectrometer (Thermo, Bremen, Germany). Each fraction was reconstituted in eluent buffer A (2% ACN, 0.1% FA) and centrifuged at 20 000×g for 10 min. Then, 10 μL supernatant was loaded onto a Shimadzu LC-20AD NanoHPLC (by the autosampler on a 2 cm C18 trap column (inner diameter 200 μm) and the peptides were eluted onto a resolving 10 cm analytical C18 column (inner diameter 75 μm). The samples were loaded at 15 μL/min for 4 min, and a 44 min gradient was then performed at 400 nL/min from 2% to 35% buffer B (98% ACN, 0.1% FA), followed by a 2 min linear gradient to 80%. The sample was maintained at 80% buffer B for 4 min and finally to 2% in 1 min.

The peptides were subjected to Nano electrospray ionization followed by tandem mass spectrometry (MS/MS) in an LTQ Orbitrap Velos coupled with HPLC. Intact peptides were detected in the Orbitrap at a resolution of 60 000. Peptides were selected for MS/MS using the high energy collision dissociation operating mode with a normalized collision energy setting of 45%. Ion fragments were detected using the Orbitrap FT-FT method. The electrospray voltage applied was 1.5 kV. Automatic gain control was used to prevent overfilling of the ion trap; 1×10^4^ ions were accumulated in the ion trap to generate of high energy collision dissociation spectra. For MS scans, the m/z scan range was 350 to 2, 000 Da.

### Protein identification and quantification

Mascot software (Matrix Science, London, UK) was used to simultaneously identify and quantify proteins. For protein identification, data files from the LC-ESI-MS/MS were searched against the NCBI Viridiplantae database (932, 602 sequences). The search parameters were as follows: trypsin was chosen as the enzyme with one missed cleavage allowed; fixed modifications of carbamidomethylation at Cys; variable modifications of oxidation of Met and N-term Glu-pyroglutamic acid; peptide mass tolerance was set at 10 ppm and fragment mass tolerance was set at ± 0.05 Da. For relative protein quantification, proteins were selected for further analysis based on the following criteria: at least two confident unique peptides, CV between the replicates smaller than 0.30. For each protein meeting the criteria between the biological and technical replicates, the iTRAQ ratios were averaged. For peptides matching multiple proteins during the database search, we conducted the quantitation using unique peptides. Proteins with average ratios greater than 2.0 were regarded as differentially altered proteins. For the gene ontology term enrichment test, the agriGO web service (http://bioinfo.cau.edu.cn/agriGO/index.php) was used [15].

### Functional analysis of salt-responsive proteins in yeast

The corresponding gene sequences of the proteins were obtained by searching the MaizeSequence database (http://ensembl.gramene.org/Zea_mays/Info/Index). The ORFs were amplified by PCR from a maize cDNA library and cloned into the yeast expression vector pYES2, which contained the Ura3 selection marker. The exogenous gene was driven by the *GAL1* promoter. The construct was introduced into yeast strain YPH500 (*ura3-52 lys2-801* ^*amber*^ *ade2-101* ^*ochre*^ *trp1-Δ63 his3-Δ200 leu2-Δ1*) according to the pYES2 vector kit instructions (Invitrogen, Carlsbad, CA, USA). Yeast salt tolerance assays were performed according to Gao et al. [16]; the NaCl concentrations were 0 M, 3 M, and 5 M. Yeast cells were collected before spotted on the agar plates, and yeast total RNAs were extracted using the RiboPure-Yeast RNA Isolation Kit (Life Technologies, Carlsbad, CA, USA).

## Results and Discussion

### Phenotypic differences between F63 and F35 under salt stress

To explore variations in salt tolerance, 162 maize inbred lines were preliminarily screened in soil compartments. The results were then validated with a hydroponic system in which seedlings harboring three full leaves were treated with 160 mM NaCl for 10 days. The two approaches produced similar results, i.e., lines F63 and F35 were the most salt-tolerant and salt-sensitive genotypes, respectively (S1 Fig.). Under a hydroponic screening system, F35 seedlings gradually withered and died while F63 seedlings survived during the 10-day of salinity treatment. The results showed that inbred line F63 was much more tolerant to salt stress than F35 at the early seedling stage. We chose these lines for further study.

**Fig 1:**
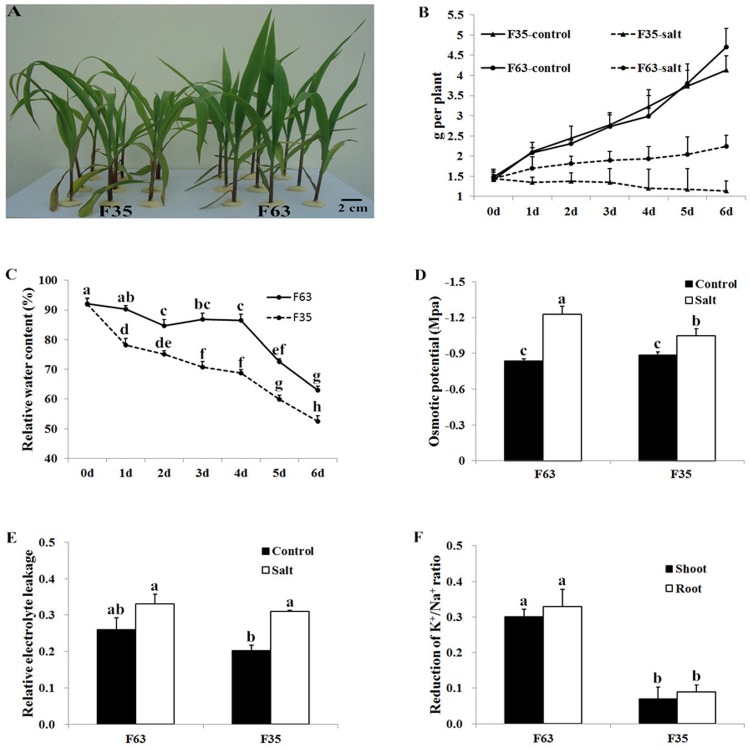
Morphological and physiological changes in F63 and F35 seedlings under NaCl stress. (A) Maize inbred lines F63 and F35 were grown hydroponically and treated with 160mM NaCl for 2 days. (B) Shoot fresh weight of F63 and F35 with or without 160mM NaCl treatment. (C) Leaf RWC of F63 and F35. (D) Leaf osmotic potential of F63 and F35 treated with 160mM NaCl for 2 days. (E) Leaf REL of F63 and F35. (F) Reduction of the K^+^/Na^+^ ratio after a 2-day NaCl treatment. For each parameter, ten seedlings were selected, and five independent biological replicates were conducted. Bars represent means ± SD (n = 5). Significant differences at P<0.01 according to Tukey’s test are indicated by different letters.

Plant growth is a classic index used to evaluate plant tolerance to abiotic stress [17–19]. Compared with plants grown under normal conditions, plants of both lines treated with 160mM NaCl showed growth retardation. However, F63 plants exhibited better growth status, with straighter and greener leaves than those of F35 (Fig. 1A, Fig. 1B). For F63, the shoot fresh weight increased gradually during the treatment, while for F35, no significant changes were observed during the first 3 days of treatment, followed by a decrease. The differences in shoot fresh weight between F63 and F35 were presumably caused by different levels of water loss in leaves under salt conditions, with F35 exhibiting sharp water loss (Fig. 1B, Fig. 1C).

Salt stress can cause both osmotic stress and ion toxicity in plants [4]. To explore the different effects of salinity stress on F63 and F35, we measured the osmotic potential, REL, and K^+^/Na^+^ ratios after a 2-day NaCl treatment. The leaf osmotic potential declined in both inbred lines under salt stress, but the rate of decline in F63 was much greater than that of F35 (Fig. 1D). The greater decline of osmotic potential might enable F63 to retain more water in response to salt stress. REL is an indicator of membrane damage under stress conditions [20]. After a 2-day salt treatment, REL increased in both F63 and F35 (Fig. 1E). However, the increase was much greater in F35 than in F63, indicating more severe membrane damage in F35. To further understand the physiological status of the maize seedlings, we determined the concentrations of Na^+^ and K^+^ in shoots and roots. Compared to the control, the Na^+^ concentrations significantly increased in both inbred lines under salt treatment. However, the rate of increase was much greater in the salt-sensitive genotype F35 than in the salt-tolerant genotype F63, indicating that the salt-sensitive genotype accumulated more Na^+^ ions after salt treatment. Growth retardation of plants under salt stress is primarily caused by the uptake of excess Na^+^, and greater accumulation of Na^+^ disruptes K^+^ absorption and inhibites the activities of many enzymes in the cytoplasm, which impairs metabolism [21,22]. In the current study, the addition of NaCl to the nutrient solution reduced the K^+^ concentration in the plant, with the greatest decline detected in the roots of F35. Although the results showed that the K^+^/Na^+^ ratio in both shoots and roots decreased after treated with 160 mM NaCl, the salt-tolerant genotype F63 exhibited less reduction in this ratio in both shoots and roots compared to F35 (Fig. 1F). Therefore, the high salt tolerance capability of F63 may be closely related to the maintenance of ion homeostasis and membrane integrity under salt stress.

The results revealed that high concentrations of NaCl increased REL and reduced the shoot fresh weight, RWC, osmotic potential, and K^+^/Na^+^ ratio in maize. However, the degrees to which maize plants reacted to salt stress were different in F63 and F35. Under NaCl treatment, F63 exhibited a smaller increase in REL and less of reduction in the K^+^/Na^+^ ratio than F35. The low osmotic potential of F63 enabled itself to retain more water and a high RWC than F35. Therefore, F63 exhibited a better growth status and higher shoot fresh weight than F35 under salt stress.

### Identification of salt stress-responsive proteins by iTRAQ LC-MS

To identify salt stress altered proteins in maize roots, we conducted a comparative proteomic analysis between F35 and F63. We extracted total root proteins from NaCl-treated and untreated seedlings and subjected them to proteomic analysis using a gel-free labeling approach. Two independent biological and technical replicates were performed using iTRAQ labeling followed by HPLC-MS/MS. We detected 856 and 857 proteins from two technical replicates, respectively (S2 and S3 Tables). The overlap of the detected proteins was summarized in S2 Fig. For the 617 reproducibly identified proteins in the replicates, 410 and 415 proteins could be quantified with at least two confident unique peptides and CV between the replicates smaller than 0.30 in F63 and F35, respectively. The detailed information of the quantified proteins was summarized in S4 Table. Compared with the control, treatment with 160 mM NaCl resulted in twenty-four and six differentially responsive proteins (>2.0 fold) in F63 and F35, respectively (Table 1). This result indicates that more proteins were altered in the salt-tolerant inbred line F63 under salt stress, suggesting that a dynamic metabolic process takes place in these plants in response to salt stimulus to help them cope with the osmotic stress and ion toxicity caused by salt stress [4].

**Table 1:** Classification of the salt-responsive proteins according to their abundance variation under salt treatment.

	F63-increased	F63-decreased	F63-constant	F63-null	Sum
F35- increased	2	0	3	1	6
F35- decreased	0	0	0	0	0
F35-constant	5	16	-	-	21
F35-null	1	0	-	-	1
Sum	8	16	3	1	28

### Classification of salt-responsive proteins

**Fig 2:**
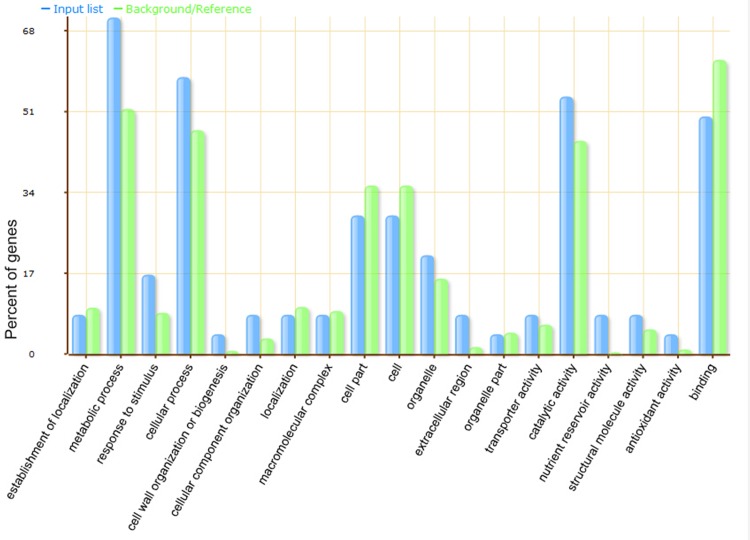
Functional classification of differentially-expressed proteins identified in this study. AgriGO web-based program was used to analyze GO categories. The X-axis is the categories of GO terms. The Y-axis is the percentage of proteins mapped by the categories. The blue column represents input (the 28 differentially-expressed proteins, N = 24). The green column represents background (maize genome reference, N = 39203).

It is of fundamental importance to identify proteins that are differentially altered under salt stress. To further characterize these proteins, we performed gene ontology (GO) analysis using the agriGO web-based program. The salt-responsive proteins were classified into a diverse category (Fig. 2). Proteins involved in carbohydrate metabolic process were specifically enriched in the metabolic process category (p-value = 8.3×10^–5^, FDR < 0.05, S5 Table). They were exhydrolase II isoform 1 (gi|162463832), fructokinase-2 (gi|162460525), xyloglucan endotransglycosylase homolog (gi|162460193), alpha-1,4-glucan-protein synthase (gi|162463414), fructose bisphosphate aldolase (gi|255645227), lichenase-2 precursor (gi|195629642) and sucrose synthase (gi|459895). No proteins were enriched in the rest of the categories.

### Differentially-regulated salt-responsive proteins in F63

As shown in Table 1, among the twenty-eight salt-responsive proteins, twenty-four were significantly altered in abundance under salt stress in salt-tolerant inbred line F63. Among the twenty-four significantly-altered proteins, twenty-two were specifically regulated in line F63. These differentially-regulated proteins might be the major contributors to the tolerant phenotype of F63.

In plants, the salt overly sensitive (SOS) signaling pathway regulates intracellular sodium ion (Na^+^) homeostasis and salt tolerance. In *Arabidopsis*, 14-3-3 proteins could inhibit the SOS signaling pathway by interacting with SOS2 protein for plant adaption to salt stress [23]. In crops, it was reported that the levels of 14-3-3 proteins were decreased in maize and rice [12,24], but increased in wheat and sugar beet under salinity stress conditions [25,26].These results indicated that 14-3-3 proteins may have diverse regulatory effects in plants in response to salt stress [8]. Here, we detected two 14-3-3 like proteins, gi|195635799 and gi|226507586, both of which were salt-reduced in F63. The decreased abundance of these two proteins in F63 might alleviate the inhibition of SOS signaling pathway, which could lead to sodium sequestration from the cytosol [27], and therefore maintain the K^+^/Na^+^ ratio better and enhanced the salt-tolerance of F63. Interestingly, the abundance of these two 14-3-3 like proteins was not changed in F35 under salt stress conditions.

Water uptake and flow across the cell membrane are essential for plant growth and development under salt stress. The plasma membrane intrinsic protein (PIP) is a subfamily of aquaporins comprising two subgroups of PIP1 and PIP2; and PIP2 proteins exhibit higher water channel activity [28]. In barley, expressions of several *PIPs* were down-regulated after 200 mM NaCl treatment, probably to prevent dehydration during salt stress [29]. In maize, *ZmPIP2-4* were salt-induced after 2 h of 100 mM NaCl treatment, and salt-reduced under 200 mM NaCl for 24 h [30]. In the present study, the abundance of maize aquaporin PIP2-4 (gi|162459653) protein decreased in F63 under 160 mM NaCl for 2 days, while remained unchanged in F35. As a result, the diffusion of water to the outside of the plasma membrane might be reduced more successfully in F63 than that of in F35 by this change. And this could make F63 exhibit a relatively higher RWC than F35 under salinity condition, and then help the plant resist the physiological drought caused by osmotic stress.

Protein synthesis is of critical importance for plant abiotic stress adaption. The levels of many components of the protein synthesis machinery are altered under salinity conditions. The abundance of most proteins that were involved in protein synthesis is reduced in *Arabidopsis* roots under salinity treatment [10]. Ribosomal proteins, an important component of protein synthesis machinery, are salt-reduced in *Arabidopsis* and maize [11,31]. In the present study, two ribosomal-related proteins, ribosomal protein S8 (gi|968902) and 60S ribosomal protein L3-1 (gi|166858), were salt-reduced in the salt-tolerant genotype F63, while they showed inverted trend, even not significant in abundance in the salt-sensitive genotype F35 (Table 2). These results indicated that, under salt stress, the salt-tolerant genotype had the ability to reduce the synthesis of redundant proteins, which may help the plant save energy to battle salt stress.

**Table 2:** Identification of Salt-Responsive Proteins in Maize Roots.

Accession‡	Description	Species	Mass§	Fold change¶		CV#	
				F63	F35	F63	F35
gi|195635409	Histone H4	*Zea mays*	24379	0.24	1.20	0.13	0.18
gi|162460024	GST-4	*Zea mays*	27768	0.26	0.71	0.25	0.17
gi|21263612	Formate dehydrogenase	*Hordeum vulgare subsp. vulgare*	50265	0.28	1.02	0.23	0.11
gi|166858	60S ribosomal protein L3–1	*Arabidopsis thaliana*	59670	0.31	1.42	0.19	0.10
gi|22160	Adenine nucleotide translocator	*Zea mays*	43216	0.33	0.81	0.07	0.08
gi|224031309	Adenosylhomocysteinase	*Zea mays*	65444	0.33	0.81	0.23	0.05
gi|293336485	Heat shock protein 90	*Zea mays*	104965	0.37	1.01	0.12	0.04
gi|226500532	Seed maturation protein PM41	*Zea mays*	56879	0.39	0.73	0.18	0.08
gi|162463414	Alpha-1,4-glucan-protein synthase	*Zea mays*	50209	0.41	1.47	0.12	0.07
gi|556673	Heat-shock protein	*Secale cereale*	108178	0.44	1.12	0.06	0.12
gi|162459653	Aquaporin PIP2–4 plasma membrane integral	*Zea mays*	33877	0.44	0.59	0.26	0.08
gi|195635799	14-3-3-like protein	*Zea mays*	36764	0.46	0.90	0.21	0.16
gi|968902	Ribosomal protein S8	*Oryza sativa Japonica Group*	33047	0.47	1.31	0.02	0.24
gi|459895	Sucrose synthase	*Zea mays*	106574	0.48	0.74	0.08	0.12
gi|162460525	Fructokinase-2	*Zea mays*	42551	0.48	0.58	0.11	0.15
gi|226507586	14-3-3-like protein	*Zea mays*	35349	0.49	0.94	0.14	0.25
gi|125558097	Hypothetical protein OsI_25768	*Oryza sativa Indica Group*	18537	0.75	2.53	0.19	0.08
gi|162460800	Peroxidase 42 precursor	*Zea mays*	36241	0.95	2.09	0.21	0.13
gi|281398970	Pathogenesis-related protein 10	*Zea mays*	20407	1.78	2.60	0.15	0.06
gi|255645227	Fructose bisphosphate aldolase	*Arabidopsis thaliana*	47373	-	2.36	-	0.12
gi|162463832	Exhydrolase II isoform 1	*Zea mays*	75783	2.54	2.01	0.14	0.19
gi|226508498	Hypothetical protein	*Zea mays*	42827	2.78	2.23	0.11	0.12
gi|162460193	Xyloglucan endotransglycosylase homolog	*Zea mays*	27232	2.05	1.29	0.22	0.20
gi|215769184	Unnamed protein product	*Oryza sativa Japonica Group*	72725	2.07	-	0.28	-
gi|226501030	hypothetical protein LOC100272932	*Zea mays*	27232	2.25	1.06	0.15	0.09
gi|76574402	Cysteine protease Mir1	*Zea diploperennis*	29993	2.39	0.67	0.06	0.10
gi|195629642	Lichenase-2 precursor	*Zea mays*	36583	3.12	0.53	0.09	0.13
gi|75994608	Cysteine protease Mir1	*Zea mays subsp. parviglumis*	30325	4.62	1.34	0.04	0.15

‡ Protein accession number from NCBInr database.

§ Protein molecular weight.

¶ Mean of protein fold changes from salt-treated samples compared with the control.

# Coefficient of variation.

In this study, we also found several proteins do not fall in any known salt stress process. For example, two cysteine proteases (gi|76574402 and gi|75994608) were salt-increased in the salt-tolerant genotype F63, but they showed no significant changes in the salt-sensitive genotype F35. These two proteins are homologs of maize insect resistance 1 (Mir1), a papain-like cysteine protease. It was reported that Mir1 rapidly accumulated in the whorls of insect-resistant maize genotypes in response to feeding by lepidopteran larvae [32,33], indicating that plants may have developed cross-tolerance mechanisms to cope with abiotic and biotic stresses [34].

### Functional verification of salt-responsive proteins in yeast

**Fig 3:**
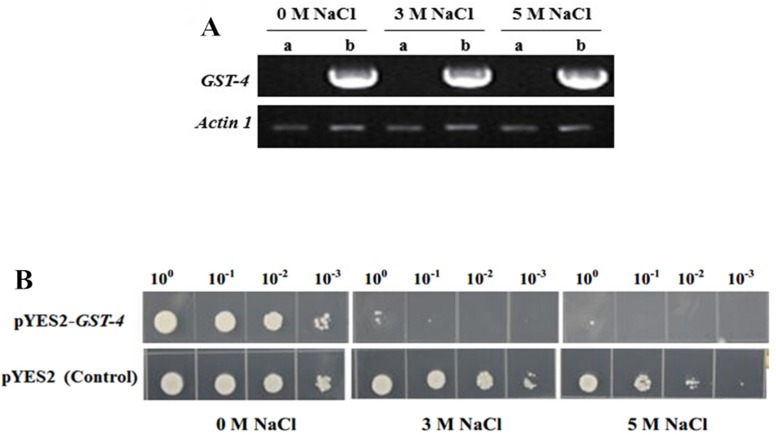
Yeast cells over-expressing *GST-4* showed decreased growth rate under salt stress. (A) Reverse-trnscript PCR analysis of *GST-4* in transgenic yeast after treated with NaCl for 24 h. a: yeast harboring pYES2 vector; b: yeast harboring pYES2-*GST-4* vector. (B) Growth of yeast cells harboring pYES2-*GST-4* vector or pYES2 vector under NaCl treatment. Yeast cells harboring pYES2-*GST-4*, or empty pYES2 vector (control) were respectively incubated in SC-ura liquid medium containing with 2% (w/v) galactose for 24 h at 30°C then adjusted to OD_600_ at 2.0 in 1 mL of medium for the stress experiments. For salt stress treatment, the yeast was resuspended in 0 M, 3 M or 5 M NaCl for 24 h. Serial dilutions were spotted onto SC-ura agar plates and incubated at 30°C for 48 h.

Many cellular processes/mechanisms of NaCl tolerance are conserved in yeast and plant cells [35,36]. In salt-tolerance genotype F63, the abundance of GST-4 (gi|162460024) decreased under salt stress. We over-expressed *GST-4* in yeast to test its role in cellular salt tolerance. RT-PCR analysis showed that *GST-4* had been transcribed in yeast under 0 M, 3 M and 5 M NaCl treatment (Fig. 3A). Over-expression of this gene did not affect the growth of yeast cells under normal conditions. However, under 3 M or 5 M NaCl treatment, yeast cells harboring pYES2-*GST-4* grew more slowly than the control (harboring empty pYES2 vector) (Fig. 3B), indicating that over-expression of *GST-4* may have a negative effect under salt stress. The results may be useful for further studies of salt-tolerance in maize.

## Conclusions

In this study, we identified twenty-eight salt-responsive proteins. Among them, twenty-two were specifically regulated in F63 but remained unchanged in F35. These proteins were mainly involved in signal processing, water conservation, protein synthesis and biotic cross-tolerance. They may contribute to the salt tolerance of this genotype. F63 exhibited a smaller increase in REL and less of reduction in the K^+^/Na^+^ ratio under NaCl treatment than F35, demonstrating that this salt-tolerant inbred line maintains membrane integrity and ion homeostasis more successfully than line F35. The low osmotic potential enabled F63 to retain more water and a high RWC in response to salt treatment. Therefore, F63 exhibited a better growth status than F35 under salt stress.

## Supporting Information

S1 Fig Maize salt tolerance screening under soil compartment and hydroponic conditions. (A) A total of 162 inbred lines were screened in salt soil pools containing 0.3% (w/v) NaCl in Nanpi County, Hebei Province, China. Fifteen seeds were sown per inbred line, and three replicates were conducted. (B) Maize inbred lines were screened under hydroponic conditions. The salt-tolerant genotype F63 and the salt-sensitive genotype F35 are indicated in the photograph.
(TIF)

S2 Fig Venn diagrams of the detected proteins in two technical replicates. Replicate I and replicate II detected 856 and 857 proteins, respectively. 617 proteins were reproducibly identified.
(TIF)

S1 Table List of 162 maize inbred lines used in this study. (XLSX)

S2 Table The proteins detected in technical replicate I. (XLS)

S3 Table The proteins detected in technical replicate II. (XLS)

S4 Table Description of the quantified proteins in F63. (XLS)

S5 Table Detail information of GO analysis for salt-responsive proteins through the agriGO program. (XLSX)
