# Supplementary figures and images for: Proteomic Analysis of Seedling Roots of Two Maize Inbred Lines That Differ Significantly in the Salt Stress Response
Source: PLoS One. 2015 Feb 6;10(2):e0116697. doi: 10.1371/journal.pone.0116697 (PMC4320067; doi:10.1371/journal.pone.0116697)

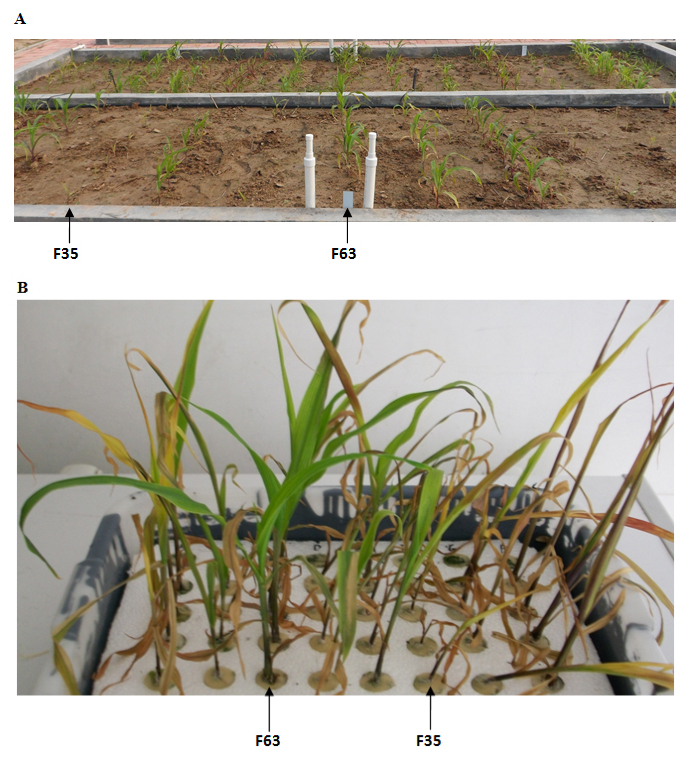

Supplement: S1 Fig — (A) A total of 162 inbred lines were screened in salt soil pools containing 0.3% (w/v) NaCl in Nanpi County, Hebei Province, China. Fifteen seeds were sown per inbred line, and three replicates were conducted. (B) Maize inbred lines were screened under hydroponic conditions. The salt-tolerant genotype F63 and the salt-sensitive genotype F35 are indicated in the photograph. (TIF) [file pone.0116697.s001.tif]

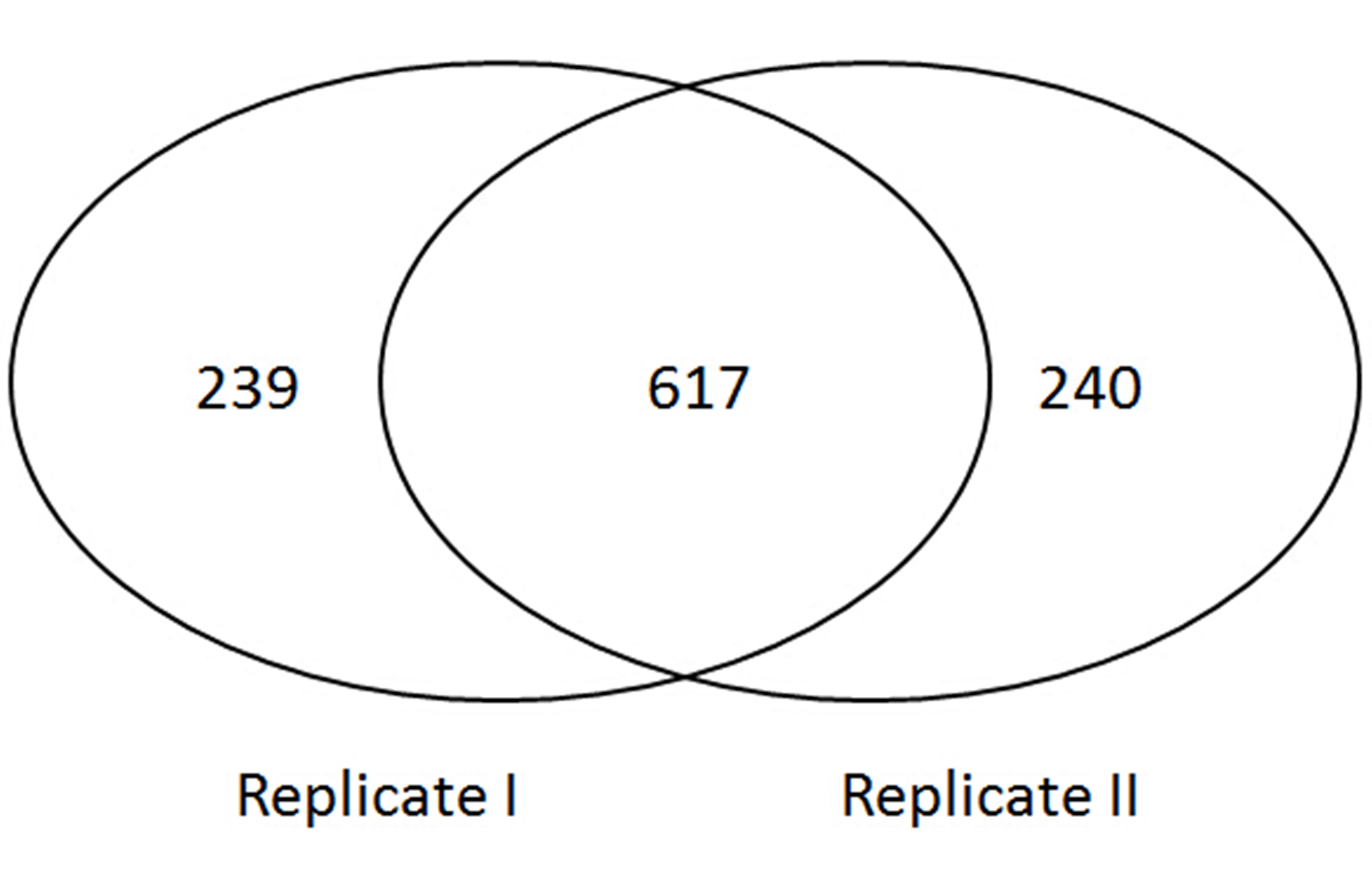

Supplement: S2 Fig — Replicate I and replicate II detected 856 and 857 proteins, respectively. 617 proteins were reproducibly identified. (TIF) [file pone.0116697.s002.tif]
